# Evaluation of Prospective ECG-Triggered CT Scan as a Practical Alternative to Standard Retrospective ECG-Gated Scan for Pre-TAVI Patients

**DOI:** 10.3390/jcm14030878

**Published:** 2025-01-28

**Authors:** Itshak Amsalem, Itzhak Vitkon-Barkay, Moshe Rav-Acha, Danny Dvir, Matan Elkan, Olga Pichkhadze, Naama Bogot, Fauzi Shaheen, Rafael Hitter, Boris Chutko, Michael Glikson, Jonathon Leipsic, Arik Wolak

**Affiliations:** 1 Jesselson Integrated Heart Center, Shaare Zedek Medical Center, Faculty of Medicine, Hebrew University of Jerusalem, Jerusalem 9103102, Israel; itshakam@szmc.org.il (I.A.); ravacham@szmc.org.il (M.R.-A.); ddvir@szmc.org.il (D.D.); fauzish@szmc.org.il (F.S.); rafaelhi@szmc.org.il (R.H.); mglikson@szmc.org.il (M.G.); 2 Cardiology Division, Shamir Medical Center, Zerifin 7033001, Israel; ivitkon@gmail.com (I.V.-B.); matanelkan@gmail.com (M.E.); 3 Department of Radiology, Shaare Zedek Medical Center, Faculty of Medicine, Hebrew University of Jerusalem, Jerusalem 9103102, Israel; olgapi@szmc.org.il (O.P.); bogotn@szmc.org.il (N.B.); 4 Department of Cardiology, Bnai Zion Medical Center, Haifa 3478403, Israel; borisch@szmc.org.il; 5 Department of Radiology and Medicine (Cardiology), University of British Columbia, Vancouver, BC V5Z 1M9, Canada; jonathon.leipsic@ubc.ca

**Keywords:** computed tomography, transcatheter aortic valve implantation, aortic stenosis, valvular heart disease

## Abstract

**Purpose:** CT-TAVI is a critical component of pre-TAVI assessment. The conventional method, retrospective ECG-gated scan, covering a complete cardiac cycle, measures the annulus during optimal systolic phases. Recently, prospective ECG-triggered scans acquiring images at a specific interval of the cardiac cycle were evaluated, allowing faster acquisition and lower contrast doses. Moreover, these scans might be beneficial for elderly patients, reducing the need for breath-holding and easing cooperation requirements. Still, their impact on annular measurement and procedural success has yet to be fully evaluated. **Methods:** This retrospective, single-center study included 419 patients who underwent CT-TAVI scans, by either prospective or retrospective scanning methods. Baseline data and calculated surgical risk scores were collected, with propensity score matching performed, followed by univariate analysis, Cox regression, and multivariable regression analysis. **Results:** A total of 171 patient pairs were generated via propensity score matching, ensuring that both groups had similar distributions of age (81 ± 8 years), sex (55% males), and baseline comorbidities. The patients in the prospective ECG-triggered group were exposed to a smaller amount of contrast material (40.0 ± 12 mL vs. 70.0 ± 48 mL, *p* < 0.001) and radiation (4.4 ± 3.6 mSv vs. 8.0 ± 10.3 mSv, *p* < 0.001). The prospective ECG-triggered group had a smaller aortic annulus area and diameter (426.6 ± 121.0 mm^2^ vs. 469.1 ± 130.8 mm^2^, *p* = 0.006 and 23.3 ± 3.2 mm vs. 24.5 ± 3.6 mm, *p* = 0.004) but no excess paravalvular leak was observed. Multivariable analysis showed no significant differences in mortality and composite endpoints between the two groups after 23 months of follow-up. **Conclusion:** Prospective ECG-triggered, ultra-fast, low-dose, high-pitch scan protocol, used in selected patients offers comparable safety and clinical procedural outcomes along with time and contrast savings.

## 1. Introduction

Cardiac CT is the preferred modality used for a thorough pre-transcatheter aortic valve implantation (TAVI) structural assessment for patients presenting with severe symptomatic aortic stenosis (AS) who are considered inoperable or at high surgical risk [1,2,3].

Recent studies emphasize the importance of precise imaging protocols to reduce radiation and contrast exposure while maintaining diagnostic quality [4]. Comprehensive assessment of the aortic annulus is crucial for selecting the appropriate valve type and size, as oversizing may lead to annulus rupture or conduction abnormalities, and undersizing may result in paravalvular leak (PVL), or, in rare cases, valve dislocation [5,6,7].

Due to aortic annulus conformational changes, pre-TAVI CT guidelines recommend a retrospective ECG-gated acquisition [3,8,9]. The term “retrospective” refers to the technique’s ability to continuously acquire images throughout the entire cardiac cycle while recording the ECG signal, allowing optimal cardiac phase selection for annulus measurement after data acquisition. This method ensures high image quality but involves significant radiation exposure and contrast medium (CM), which might be problematic for renal-impaired patients. Furthermore, this scanning method often requires a breath hold, which can be challenging for the elderly [10,11,12,13].

In contrast, the prospective ECG-triggered, ultra-fast, low-dose high-pitch protocol (hereafter prospective ECG-triggered scan) refers to a forward-planned approach where imaging is synchronized with a specific point in the cardiac cycle, as determined by real-time ECG signals. This method offers a faster, low-contrast, and potentially low-radiation scan that can be executed without breath-hold due to its very short acquisition time [14,15,16,17,18,19,20,21,22,23]. However, this method limits the scan to one phase and does not provide full cardiac cycle data.

Given the paucity of available information regarding the clinical significance of these new protocols in evaluating TAVI candidates, the study-center TAVI database was used to investigate the feasibility, safety, and impact on TAVI outcomes of prospective ECG-triggered scans in that population.

## 2. Materials and Methods

### 2.1. Study Population

This retrospective study included patients who underwent TAVI between March 2018 and September 2021, all of whom had pre-procedure CT scans performed at Shaare Zedek Medical Center. All patients had severe AS according to current guidelines definition and were TAVI-referred by the heart team [1,2,20].

Baseline characteristics, medical history, laboratory tests, calculated surgical risk scores, and echocardiographic findings were obtained from the Israeli TAVI Multicenter Registry [24,25,26]. CT study data were acquired from a local database. Mortality data were taken from the National Population Register.

### 2.2. Scanning Protocols and Patient Selection

#### 2.2.1. CT Data Acquisition and Analysis

All images were acquired using a third-generation dual-source CT (DSCT) system (Somatom Force, Siemens Healthineers, Forchheim, Germany). Scans were scheduled with bolus tracking monitoring, with the region of interest (ROI) placed on the descending aorta (at the level of the main pulmonary artery), with a threshold of 100 Hounsfield Units (HUs). The examinations were conducted using Care kV tube potential modulation technology and reconstructed with iterative reconstruction, Admire 3.

The retrospective ECG-gated scan group was scanned using three consecutive scans. First, a preliminary non-contrast CT scan (NCCT) was conducted for aortic valve Agatston score assessment and selection of the acquisition field of view. This was followed by a retrospective ECG-gated scan using a full acquisition window (0–100% of the RR cycle) covering the heart. Image reconstructions were performed at “Best Diastole”, 30% and 35% phases. Additional reconstructions were performed every 10% (phase 0–100%). For peripheral arterial assessment, a third non-gated high-pitch scan (a “turbo-FLASH” with a pitch of 2.2) was performed, covering the area from the clavicula to the ischium. In the prospective ECG-triggered scan group, after an NCCT, a single ECG-gated prospective high-pitch scan (with a pitch of 3.2) was performed from the clavicula to the ischium. The acquisition window was set to 65% of the RR cycle. As per Siemens’ default protocol settings, these patients were not given any breath-hold instructions. The specific settings for each scanning protocol are described in Table S1 (Supplementary Materials).

#### 2.2.2. Selection of Scanning Protocol

As a default, in the study institution, candidates for TAVI undergo a retrospective ECG-gated scan before the procedure. However, in the following circumstances, a prospective ECG-triggered scan was preferred: Patients with poor renal function, fragile patients, or patients whom the cardiac imaging specialist or the CT technician estimated would have difficulty holding their breath or cooperating during the scan (such as in cases of hearing loss, back pain while supine on the gantry, language barrier, or cognitive impairment). Patients who were not able to walk without support and move independently to the scanner bed were defined as “fragile”. The cardiac imaging specialist made the final decision regarding the specific protocol.

### 2.3. CT Post-Processing

A cardiac imaging specialist performed a coronary assessment using Syngo.Via Workstation (Siemens Healthineers, Forchheim, Germany). Aortic root anatomy and the aorta-iliofemoral vasculature were reviewed using either syngo.Via Workstation TAVI module or 3mensio Workstation (Pie Medical Imaging, Maastricht, The Netherlands) by a cardiovascular imaging expert with over ten years of experience in CT-TAVI interpretation (AW and NB). Two senior readers independently reviewed and co-signed each CT report, ensuring a cross-validation of the findings. In all the patients that were included in the study, valve sizing was exclusively based on CT measurements.

### 2.4. TAVI Procedure

See the Supplementary Materials (S3) for TAVI procedure details.

### 2.5. Echocardiography

A transthoracic echocardiographic study was performed at baseline for ventricular and valvular structural assessment. Follow-up echocardiography was performed within 30 days of the TAVI procedure, mostly during the index hospitalization. Assessment of AS, aortic regurgitation (AR), and PVL was performed according to current guidelines, using the five-class grading scheme [21].

### 2.6. Study Outcomes

Primary outcome:

Overall all-cause mortality.

Secondary outcomes:

1. Composite Valve Academic Research Consortium3 (VARC-3) endpoints for technical failure, device failure, and early safety events [27]. [See the Supplementary Materials (S4) for a detailed description of the elements comprising the composite outcomes.]
2. In hospital, 30-day, and 1-year mortality.
3. Paravalvular leak (PVL)—defined as more then mild PVL.
4. Safety parameters—need for a second valve, valve malposition, in-hospital stroke/transient ischemic attack (TIA), acute kidney injury (AKI) (VARC-3, stage 1–3), major vascular complications (VARC-3), new post-procedural complete atrioventricular (AV) block, and new permanent pacemaker implantation.

### 2.7. Statistical Analysis

Descriptive statistics for studied variables are presented as mean (standard deviation, SD) for normally distributed variables, median (interquartile range, IQR) for non-normally distributed variables, and frequency (percentage) for categorical variables. Correlation between two numeric variables was performed by Pearson’s correlation method for normally distributed variables. For non-normally distributed variables, we used Spearman’s correlation test.

Comparisons between independent groups were performed by independent sample T-test for continuous variables and Chi-square test for categorical variables. For non-normally distributed continuous variables, we used the Independent-samples Mann–Whitney U Test.

Univariate and multivariable regression analyses were used to measure the strength of the relationship between VARC-3 composite outcomes and studied variables. Analysis of event-time (survival) data, including hazard ratios (HRs) and confidence limits, was calculated using the Cox proportional hazards model.

Power calculation—the overall 1-year mortality after TAVI is approximately 10%, as was shown in several studies [26,28]. The only study, to our knowledge, that examined mortality in TAVI patients who were evaluated by prospective ECG-triggered CT scan showed 14% mortality at its lowest [29]. Based on these studies, a sample of 322 patients (161 in each group) will be sufficient to exclude a difference in favor of retrospective ECG-gated CT scans greater than 5% with a power of 80% and a significance level of 5%. All analyses were conducted using SPSS statistical software (version 26.0, SPSS Inc., Chicago, IL, USA). All statistical tests were two-sided, and significance was determined at a *p*-value of 0.05.

## 3. Propensity Score Matching

To adjust for the given selection bias in choosing the CT method and to minimize confounding by indication, which is mainly accounted for by renal function, we used propensity score matching (PSM).

Using the built-in PSM in SPSS version 26 we have divided the cohort into CT groups by matching to each patient’s glomerular filtration rate (GFR). Match tolerance was set at 0.01 to maximize accurate matching.

## 4. Results

### 4.1. Patient Characteristics

Matching resulted in 171 patient pairs with aligned characteristics, minimizing potential confounders. Mean ages were 81.1 ± 7.8 and 80.1 ± 7.8 years in the prospective ECG-triggered scan group and the retrospective ECG-gated scan group, respectively. Regarding sex distribution, the prospective ECG-gated and retrospective ECG-gated groups had 52.6% and 55.6% males, respectively. Other clinical characteristics and comorbidities, including coronary artery disease (CAD), chronic kidney disease (CKD), peripheral vascular disease (PVD), diabetes mellitus, chronic obstructive pulmonary disease (COPD), New York heart association (NYHA) class, II, and STS-scores, were balanced between the two groups (Table 1) (for additional descriptive information of the unmatched population, see Table S2 in the Supplementary Materials).

### 4.2. CT and Procedural Data

Patients in the retrospective ECG-gated scan group were exposed to more contrast (70.0 ± 48 mL vs. 40.0 ± 12 mL, *p* < 0.001) and more radiation (effective dose 8.0 ± 10.3 mSv vs. 4.4 ± 3.6 mSv, *p* < 0.001) as compared with patients in the prospective ECG-triggered scan group. Measured aortic valve annulus diameter and aortic valve annulus area were larger in the retrospective ECG-gated scan group (24.5 ± 3.6 mm vs. 23.3 ± 3.2 mm, *p* = 0.004, and 469.1 ± 130.8 mm^2^ vs. 426.6 ± 121.0 mm^2^, *p* = 0.006) and bigger prosthetic valves were used in this group (26.5 ± 2.8 mm vs. 25.6 ± 2.4 mm, *p* = 0.001). Technical quality was comparable, a part of a significantly higher percent of severe artifacts, at the annulus-LVOT level, in the prospective ECG-triggered group (Table 2). Severe artifacts were defined as artifacts that obscured anatomic details and hindered the interpretation of the CT angiography study. S5 in Supplementary Materials provides detailed data regarding image quality analysis.

### 4.3. Mortality

Mortality follow-up time ranged between 1.5 and 4.5 years, with a median follow-up of 23 months. There were no significant differences between the propensity-matched groups for in-hospital, 30-day, 1-year, and overall mortality rates in the prospective ECG-triggered scan group compared to the retrospective ECG-gated scan group (1.6% vs. 1.2% *p* = 0.65; 1.8% vs. 1.8%, *p* = 1.0; 7.0% vs. 5.8%, *p* = 0.66; 15.7% vs. 12.3%, *p* = 0.2, respectively) (Table 3).

**Figure 1:**
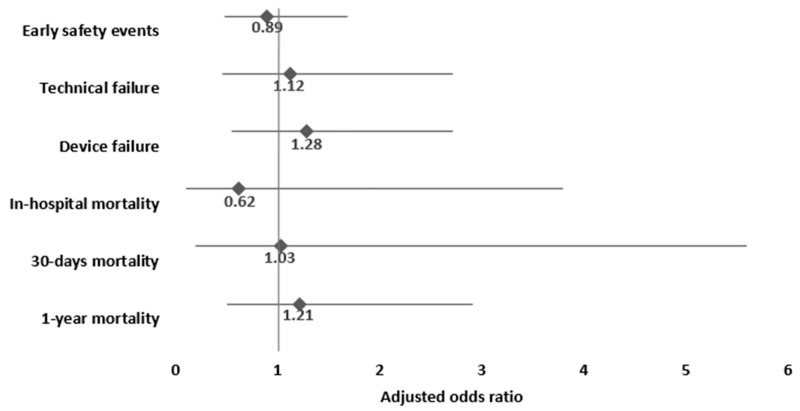
Multivariable adjusted analysis for the associations of prospective ECG-triggered scan, compared to retrospective ECG-gated scan (reference category), and study outcomes. Footnote: adjusted for sex, age, EUROSCORE II, and chronic kidney disease.

Multivariable analysis for the study outcomes adjusted for sex, age, EUROSCORE II, and CKD yielded no significant difference between the prospective ECG-triggered scan group as compared to the retrospective ECG-gated scan group (serving as reference) with ORs for in-hospital, 30-day, 1-year, and overall mortality of 0.62, CI 0.1–3.8; 1.03, CI 0.19–5.56; 1.21, CI 0.5–2.91; and 1.56, CI 0.85–2.87, respectively (Figure 1).

Time-to-event analysis of survival in the prospective ECG-triggered scan group compared to the retrospective ECG-gated scan group, adjusted for sex, age, EUROSCORE II, and CKD showed a non-significant hazard ratio of 1.45 (CI 0.82–2.53).

### 4.4. Procedural and Technical Outcome

There was no significant difference between the groups for VARC-3 composite endpoints for technical or device failures after PSM. In a multivariable regression analysis adjusted for sex, age, EUROSCORE II, and CKD no significant differences were found in the prospective ECG-triggered scan group compared to the retrospective ECG-gated scan group in technical failure (OR 1.12, CI 0.46–2.72) and device failure (OR 1.28, CI 0.55–2.72).

Procedural complications, including valve malposition and second valve implantations, were low, with no significant difference between the two groups after PSM (Table 3). Open surgery or emergency bypass surgery was unnecessary in any of the cases. There was no coronary obstruction, ventricular septal perforation, cardiac tamponade, annular rupture, or left ventricular outflow tract (LVOT) obstruction in the study population.

### 4.5. Clinical Safety Outcomes

Early safety events during hospitalization were comparable in the prospective ECG-triggered scan group and retrospective ECG-gated scan group before and after PSM (14% vs. 12.9%, respectively, *p* = 0.75). Multivariable analysis showed no significant difference in early safety events between the prospective ECG-triggered scan group and the retrospective ECG-gated scan group (OR 0.89, CI 0.48–1.68) (Figure 1).

Stroke rates were comparable among the prospective ECG-triggered scan group as compared to the retrospective ECG-gated scan group (1.8% vs. 2.4%, respectively, *p* = 0.9). Major vascular complications rates did not differ between the prospective and retrospective scan groups (4.1% vs. 1.2%, *p* = 0.24), nor did acute AKI rates (1.8% vs. 1.2%, respectively, *p* = 0.6). There were similar rates of high-degree conduction disorders requiring pacemakers (8.2% vs. 7.6%, respectively, *p* = 0.8).

Overall PVL rates did not differ significantly between the prospective and retrospective ECG-triggered scan groups (7.1% vs. 10.5%; *p* = 0.6). Additionally, 3.1% of the prospective ECG-triggered scan group had mild or moderate PVL at follow-up echocardiography vs. 6.3% in the retrospective ECG-gated scan group (*p* = 0.31). No severe PVL was documented in either group.

## 5. Discussion

This study shows no significant difference in mortality, technical failure, device failure, and early safety events between the ECG-gated prospective high-pitch and the standard retrospective ECG-gated scans. Differences found included minimal (but statistically significant) variations in annular sizing and a lower rate of significant severe artifacts in the prospective ECG-triggered scan. Notably, patients in the prospective ECG-triggered scan group were exposed to a significantly lower amount of CM and radiation than those in the retrospective ECG-gated scan group.

This study was driven by a practical need to optimize pre-TAVI CT scans, particularly for elderly patients, many of whom appeared frail, often had renal dysfunction, and were likely to struggle with breath-holding instructions required in the standard protocol. Performing a retrospective ECG-gated scan in patients with limited cooperation may result in significant motion artifacts, rendering the scan non-diagnostic not only for coronary assessment but also for precise aortic root measurements. This would necessitate repeat scans, increasing procedure time, and doubling contrast exposure, an outcome we aspire to avoid, particularly in patients with CKD. Although the impact of CM on the development of AKI after TAVI is still a matter of debate, current guidelines recommend minimizing CM use when possible [30]. Notably, even in this study, where retrospective CT scans were performed in patients who appeared capable of holding their breath, a significantly increased percentage of motion artifacts was observed compared to the prospective ECG-triggered CT scan group. Therefore, it could be reasonably inferred that the artifact rate would have been much higher had patients from the prospective CT group been referred for the standard retrospective CT scan.

Throughout the last decade, several studies investigated the technical feasibility of prospective ECG-triggered scan protocols in pre-TAVI evaluation, demonstrating that these protocols are non-inferior to standard-pitch acquisitions for aortic assessment while using less CM, reducing the amount of radiation, and permitting a no breath-hold scan due to their high pitch [17,18,19,20]. However, evaluation of the clinical impact of the prospective ECG-triggered scan method on TAVI outcomes was not performed in previous trials, and this is the main novelty and clinical relevance of the current study.

A previous study by Steffen et al. showed an excess of mortality at three-year follow-up among patients who underwent prospective ECG-triggered high-pitch systolic phase acquisitions of the aortic valve, as compared with those who had diastolic phase acquisitions of the aortic valve before TAVI [29]. However, unlike the mentioned study, our study does not compare systolic and diastolic scanning protocols but rather two different scanning methods. While the prospective ECG-triggered scan protocol does not guarantee optimal timing of the scan for the systolic phase, it saves time and CM without compromising the clinical outcomes of the TAVI procedure. These may be of value in elderly and frail patients and may enable high-quality imaging, even in less cooperative patients.

The observed reduction in respiratory artifacts in the prospective ECG-triggered group likely stems from the protocol’s key features. The ultra-fast acquisition time minimizes the likelihood of motion during the scan, even in patients who may struggle with cooperation. Furthermore, the absence of breath-hold requirements eliminates potential variability caused by inconsistent or incomplete breath control, which is particularly relevant in frail or elderly populations. These factors combined contribute to the improved image quality and explain the lower rate of artifacts in this group.

In this study, the prospective ECG-triggered scan group exhibited slightly smaller measurements for both the aortic annulus diameter and area and a smaller median size of the implanted prosthetic valves. These findings are in line with previous data [8]. The smaller annular size measured via the prospective scan has probably led to using a smaller prosthetic valve size. Importantly though, despite the smaller valve sizes used in the prospective ECG-triggered scan group, the PVL rates were not increased as compared to those found in the retrospective ECG-gated group. Several factors could explain this finding. First, unknown confounders in the prospective group, which were not balanced by the propensity match, may have contributed to smaller annular sizes. Thus, the apparently smaller annulus observed in the prospective CT group may have actually been the correct size for this group. Second, in the retrospective ECG-gated group, the aortic annular measurements (and thus prosthetic valve sizes) might have been subjected to over-sizing due to the specific phase selected for annular measurement. Although over-sizing may lead to complications such as conduction disturbances or annular rupture, a study by Elnwagy et al. found that prosthesis over-sizing by 20% was safe and effective, with no significant effect on conduction system complications or annular injury [31]. Finally, the limited sample size of the study may have reduced statistical power, preventing the detection of more subtle differences in PVL rates. Overall, while pulsatile changes in the aortic annulus could potentially affect the prosthesis sizing, the current study findings suggest that the small difference in annular sizing between the two CT scanning groups, although statistically significant, does not translate into a significant change in the TAVI clinical outcomes.

This study presents several limitations: this study is a retrospective, single-center, single-vendor study, which may limit the generalizability of the findings to centers using different scanner models or imaging protocols. While our results are based on a Siemens dual-source CT scanner, similar improvements might be achievable with alternative protocols in modern wide-detector or single-source systems. Data regarding the specific reason for using prospective over retrospective scans were unavailable, limiting the assessment of factors influencing protocol selection. An additional limitation of this study is the reliance on an informal assessment of frailty, rather than utilizing one of the established frailty scoring systems [32]. While the study reports long-term all-cause mortality, this endpoint warrants cautious interpretation since mortality in such an old and sick population can arise from multiple causes, complicating the attribution of outcomes solely to the TAVI procedure. This study did not report the phase in which the aortic root was scanned in the prospective ECG-triggered scan protocol. The scan starts from the level of the clavicles, arriving at the aortic root in some delay, the length of which depends on the patient’s height and pulse. Therefore, the exact point at which the scan reached the annulus had to be calculated individually for each patient and, even then, as an approximation only.

While the idea of a FLASH technique for pre-TAVI assessment is not new and has been previously studied, this study distinctively emphasizes the clinical relevance of this protocol based on a sizable cohort of patients. This study adds value by providing outcome data that was not thoroughly addressed in previous research.

## 6. Conclusions

Prospective ECG-gated, ultra-fast, low-dose, high-pitch scan protocol can save time and contrast while providing high-quality imaging, even in a poorly cooperative patient. Although this study suggests that it provides a similar safety profile in the short and medium term without excess adverse events such as PVLs or mortality, the decision to use this protocol should be based on careful clinical judgment. It may be a practical option for patients who struggle with breath-holding; however, its use should be performed with discretion and reserved for situations where standard protocols are not feasible.

## Figures and Tables

**Table 1:** Unmatched and propensity-matched * baseline characteristics of the study population.

	Unmatched Population				Propensity-Matched Population *		
	Total	Retrospective ECG-Gated Scan	Prospective ECG-Triggered	*p* Value	Retrospective ECG-Gated Scan	Prospective ECG-Triggered	*p* Value
N	419	191	228		171	171	
Demographics							
Age (mean (SD))	80.6 (7.8)	80 (7.8)	81 (7.6)	0.13	80.1 (7.8)	81.1 (7.8)	0.25
Male (%)	225 (53.7%)	96 (50%)	129 (56%)	0.19	90 (52.6%)	95 (55.6%)	0.58
BMI (%)	27.6 (6.2)	27.9 (5.7)	27.4 (6.6)	0.4	27.6 (5.4)	27.5 (6.9)	0.87
Medical background							
Coronary artery disease (%)	180/415 (43.4%)	79 (42%)	101 (44%)	0.67	74 (43.3%)	68 (39.8%)	0.51
Hypertension (%)	254/346 (73.4%)	121 (73%)	133 (74%)	0.83	113 (74.3%)	102 (75.6%)	0.81
Diabetes mellitus (%)	141/336 (42%)	62 (38%)	79 (45%)	0.18	59 (39.9%)	57 (42.5%)	0.65
COPD (%)	33/415 (8%)	18 (9.6%)	15 (6.5%)	0.25	18 (10.5%)	10 (5.8%)	0.11
NYHA-Class (%)				0.77			0.98
Class 2	25/415 (6%)	13 (6.9%)	12 (5.2%)		13 (7.6%)	12 (7.0%)	
Class 3	322/415 (77.6%)	144 (77%)	178 (78%)		130 (76.0%)	131 (76.6%)	
Class 4	68/415 (16.4%)	30 (16%)	38 (16%)		28 (16.4%)	28 (16.4%)	
Chronic kidney disease (%)	180 (43%)	68 (35%)	112 (49%)	0.005	63 (36.8%)	68 (36.8%)	1
Chronic dialysis (%)	8/414 (1.9%)	5 (2.7%)	3 (1.3%)	0.31	5 (2.9%)	3 (1.8%)	0.47
GFR (mL/min) (mean (SD))	64.2 (25.2)	67.2 (22.7)	61.6 (26.8)	0.02	66.5 (22.8)	66.2 (23.2)	0.89
Peripheral vascular disease (%)	14/415 (3.4%)	3 (1.6%)	11 (4.8%)	0.07	3 (1.8%)	8 (4.7%)	0.12
STS score (mean (SD))	3.05 (2.7)	3.07 (3.2)	3.03 (2.2)	0.88	3.19 (3.4)	2.88 (2.1)	0.31
EuroSCORE II (mean (SD))	5.51 (5.7)	5.11 (4.9)	5.83 (6.2)	0.19	5.34 (5.1)	5.60 (6.0)	0.66

* Matched for glomerular filtration rate (GFR). Abbreviations: SD—standard deviation; NYHA-Class—New York Heart Association Functional Classification; BMI—body mass index; COPD—chronic obstructive pulmonary disease; GFR—glomerular filtration rate; STS score—Society of Thoracic Surgeons’ score.

**Table 2:** CT and TAVI data of the propensity-matched population.

	Retrospective ECG-Gated Scan	Prospective ECG-Triggered	*p* Value
N	171	171	
CT estimated effective radiation dose (mSv), median (IQR)	8.04 (10.3)	4.42 (3.6)	**<0.001**
CT contrast (mL), median (IQR)	70.0 (48)	40.0 (12)	**<0.001**
Aortic valve annulus diameter (mm), median (IQR)	24.5 (3.6)	23.3 (3.2)	**0.004**
Aortic valve annulus area (mm^2^), median (IQR)	469.05 (130.8)	426.6 (121.0)	**0.006**
Contrast material volume (mL) during TAVI, median (IQR)	100.0 (50)	100.0 (40)	**0.014**
Artifact due to respiratory motion	13 (7.6%)	1 (0.6%)	**0.001**
Prosthetic valve size, mean (SD)	26.5 (2.8)	25.6 (2.4)	**0.001**
Prosthetic valve size by groups			**0.001**
20–24	31 (18.5%)	59 (34.5%)	
25–28	85 (50.6%)	80 (46.8%)	
29–34	52 (31.0%)	32 (18.7%)	

Abbreviations: CT—computed tomography; IQR—interquartile range; SD—standard deviation.

**Table 3:** TAVI outcomes and complications among the propensity-matched population.

	Retrospective ECG-Gated Scan	Prospective ECG-Triggered	*p* Value
N	171	171	
Mortality			
Mean follow-up (month, SD)	26.01 (14.1)	27.29 (12.5)	0.37
Procedural mortality (%)	1 (0.6%)	1 (0.6%)	1
In-hospital mortality (%)	2 (1.2%)	3 (1.6%)	0.65
30-day mortality (%)	3 (1.8%)	3 (1.8%)	1
1-year mortality (%)	10 (5.8%)	12 (7.0%)	0.66
Overall mortality (%)	21 (12.3%)	32 (15.7%)	0.2
Composite outcomes			
Technical failure (%)	10 (5.8%)	11 (6.4%)	0.82
Device failure (%)	14 (8.2%)	17 (9.9%)	0.57
Early safety events (%)	22 (12.9%)	24 (14.0%)	0.75
Paravalvular leak			
Angiographic paravalvular leak (%)			0.6
None	153 (89.5%)	158 (92.9%)	
Minimal	11 (6.4%)	6 (3.5%)	
Mild	5 (2.9%)	5 (2.9%)	
Moderate	2 (1.2%)	1 (0.7%)	
Severe	0	0	
Echocardiographic paravalvular leak (%)			0.31
None	149 (93.7%)	156 (96.9%)	
Mild	6 (3.8%)	4 (2.5%)	
Moderate	4 (2.5%)	1 (0.6%)	
Complications			
Need for a second valve (%)	3 (1.8%)	3 (1.8%)	0.99
Valve malposition (%)	3 (1.8%)	2 (1.2%)	0.65
In-hospital stroke/TIA (%)	4 (2.4%)	3 (1.8%)	0.9
Acute kidney injury (VARC-3) (stage 1–3) (%)	2 (1.2%)	3 (1.8%)	0.6
Major vascular complications (VARC-3) (%)	2 (1.2%)	7 (4.1%)	0.24
New post-procedural complete AV block (%)	13 (7.6%)	14 (8.2%)	0.8
New permanent pacemaker implantation (%)	13 (7.6%)	16 (9.4%)	0.57

Abbreviations: SD—standard deviation; NYHA-Class—New York Heart Association functional classification; BMI—body mass index; COPD—chronic obstructive pulmonary disease; GFR—glomerular filtration rate; STS score—Society of Thoracic Surgeons’ Score; TIA—transient ischemic attack; CPR—cardiopulmonary resuscitation; AV block—atrioventricular block.

## Data Availability

The original contributions presented in this study are included in the article/Supplementary Materials. Further inquiries can be directed to the corresponding authors.

## Supplementary Materials

The following supporting information can be downloaded at: https://www.mdpi.com/article/10.3390/jcm14030878/s1.
