# Supplementary material for: Evaluation of Prospective ECG-Triggered CT Scan as a Practical Alternative to Standard Retrospective ECG-Gated Scan for Pre-TAVI Patients
Source: J Clin Med. 2025 Jan 28;14(3):878. doi: 10.3390/jcm14030878 (PMC11818146; doi:10.3390/jcm14030878)
Supplement: Supplementary file 1 [file jcm-14-00878-s001.zip › jcm-3427030-supplementary.pdf]

<u>Supplementary Materials</u>

Table of contents:

Table S1– CT protocols 2

Table S2 – Characteristics of the unmatched population... 3

S3. TAVI procedure 5

S4. Composite Valve Academic Research Consortium3 (VARC-3) endpoints: 5

S5. Qualitative Image Quality Assessment... 6

Table S1: CT protocols.

**Group**	**Retrospective ECG-gated scan**<br>**2 consecutive scans**		**Prospective ECG-triggered scan**
	SPIRAL scan	High pitch scan	High pitch scan
**Acquisition field of view**	Heart	Clavicula to ischium	Clavicula to ischium
**ECG gating**	Yes	No	yes
**Acquisition window**	0-100%	n.a.	65%
**Pitch**	0.15 – 0.35<br>Heart rate dependent	2.2	3.2
**Rotation time**	0.25 sec	0.25 sec	0.25 sec
**Collimation**	192 x 0.6	192 x 0.6	192 x 0.6
**Tube potential**	Automatic<br>– CarekV on<br>– 70 – 120kV<br>– Optimized for vascular image representation	Automatic<br>– CarekV on<br>– 70 – 120kV<br>– Optimized for vascular image representation	Automatic<br>– CarekV on<br>– 70 – 120kV<br>– Optimized for vascular image representation
**Tube current**	Automatic<br>– CareDose4D on<br>– Quality Ref. mAS 288	Automatic<br>– CareDose4D on<br>– Quality Ref. mAS 174	Automatic<br>– CareDose4D on<br>– Quality Ref. mAS 288
**Contrast material volume**	Patient weight/1.8 + 15ml		Patient weight/2 (max. 45ml when GFR<45ml/min)
**Contrast material flow**	Total contrast volume/11		Total contrast Volume/9
**Delay between contrast injection and initiation of scan**	6 sec from contrast enhancement threshold (100 HUs) within the ROI		7 sec from contrast enhancement threshold (100 HUs) within the ROI
**Reconstruction**	1. At best Diastole/0.6 slice thickness/ Bv36 kernel.<br>2. At phases 30% and 35%/Bv36 kernel/0.6 slice thickness every 10% (phase 0-100%)/0.6 slice thickness/Bv36 kernel.	A full-body reconstruction/2mm slice thickness/Bv36 kernel.	1. Reconstruction of the heart using slice thickness- 0.6/ Bv36 kernel.<br>2. A full-body reconstruction/2mm slice thickness/Bv36 kernel.

Abbreviations: KV—kilovoltage; mAS—milliampere-seconds

Table S2: characteristics of the unmatched population.

	Retrospective ECG-gated scan	Prospective ECG-triggered	P value
**N**	191	228	
Composite outcomes			
**Technical failure**	11 (5.8%)	11 (4.8%)	0.66
**Device failure**	15 (7.9%)	18 (7.9%)	0.99
**Early safety events**	24 (12.6%)	29 (12.7%)	0.96
**Mean follow-up (months)**	25.2 (13.8)	26.3 (12.7)	0.4
**Procedural mortality**	1 (0.5%)	1 (0.4%)	0.9
**In-hospital mortality**	2 (1.0%)	3 (1.3%)	0.8
**30-day mortality**	3 (1.6%)	3 (1.3%)	0.82
**1-year mortality**	11 (5.8%)	20 (8.8%)	0.24
**Overall mortality**	22 (11.5%)	48 (21.1%)	0.009
CT details			
**CT estimated effective dose (mSv), median (IQR)**	8.04 (9.9)	4.4 (3.5)	<0.001
**CT contrast (ml), median (IQR)**	70.0 (50)	41.0 (12)	<0.001
**Aortic valve annulus diameter (mm), median (IQR)**	24.05 (3.6)	23.5 (3.4)	0.029
**Aortic valve annulus area (mm2), median (IQR)**	457.5 (129.1)	426.6 (122.3)	0.021
**Contrast material volume (ml) during TAVI, median IQR)**	104.5 (60)	98 (40)	0.001
**Respiratory motion**	15 (7.9%)	1 (0.4%)	<0.001
**Technical quality**			0.11
**Poor**	0 (0%)	2 (0.9%)	
**Fair**	29 (15.2%)	22 (9.6%)	
**Good**	134 (70.2%)	178 (78.1%)	
**Excellent**	28 (14.7%)	26 (11.4%)	
Prosthetic valve measurements			
**Valve size, mean (SD)**	26.3 (2.9)	25.7 (2.5)	0.04
**Valve size by groups**			0.03

**20-24**	46 (24.7%)	75 (33.3%)	
**25-28**	86 (46.2%)	107 (47.6%)	
**29-34**	54 (29.0%)	43 (19.1%)	
Paravalvular leak			
**Angiographic paravalvular leak**			0.61
**None**	206 (91.2%)	177 (93.7%)	
**Minimal**	13 (5.8%)	6 (3.2%)	
**Mild**	5 (2.2%)	5 (2.6%)	
**Moderate**	2 (0.8%)	1 (0.5%)	
**Severe**	0	0	
**Echocardiographic paravalvular leak**			0.41
**None**	202 (94.8%)	175 (97.2%)	
**Mild**	7 (3.3%)	4 (2.2%)	
**Moderate**	4 (1.9%)	1 (0.6%)	
Complications			
**Need for a second valve**	3 (1.5%)	3 (1.3%)	0.82
**Valve malposition**	3 (1.5%)	2 (0.8%)	0.51
**In-hospital ischemic stroke/TIA**	5 (2.7%)	4 (1.7%)	0.81
**Acute kidney injury (VARC-3) (stage 1-3)**	2 (1.05%)	8 (3.5%)	0.36
**Major vascular complications (VARC-3)**	3 (1.5%)	7 (3.09%)	0.51
**New post-procedural complete AV block**	13 (6.8%)	17 (7.5%)	0.63
**New permanent pacemaker implantation**	13 (6.8%)	20 (8.8%)	0.46
**Mitral valve apparatus damage or dysfunction**	1 (0.5%)	0	0.27
**Procedure CPR**	2 (1.1%)	1 (0.4%)	0.46

Abbreviations: NYHA-Class—New York Heart Association functional classification; BMI—body mass index; COPD—chronic obstructive pulmonary disease; GFR—glomerular filtration rate; STS score—Society of Thoracic Surgeons' Score; TIA—transient ischemic attack; CPR—cardiopulmonary resuscitation; AV block—atrioventricular block.

S3. TAVI procedure

- In most patients, the procedure was performed with a femoral approach.
- A decision regarding a different vascular approach was made by the Heart Team.
- The decision to use conscious sedation or general anesthesia was at the operators' discretion.
- Anticoagulation was obtained by using unfractionated heparin.
- After device deployment, an angiographic, semi-quantitative assessment of PVL was performed.
- Closure devices were used to close the access site.
- Recommendations for post-procedural antithrombotic or anticoagulant treatment were given in accordance with the relevant guidelines.

S4. Composite Valve Academic Research Consortium3 (VARC-3) endpoints

Technical failure (at exit from procedure room):

Mortality.

Failure to deliver the device.

Device malposition.

Unplanned surgery or intervention related to the device failure.

Echocardiographic parameters of high-pressure gradient across the bioprosthetic valve or more than moderate AR, at the end of the procedure and during index hospitalization, respectively.

Device failure (at 30 days or the end of index hospitalization):

Mortality.

Failure to deliver the device.

Device malposition.

Unplanned surgery or intervention related to the device failure.

Echocardiographic parameters of high-pressure gradient across the bioprosthetic valve or more than moderate AR, at the end of the procedure and during index hospitalization, respectively.

Early safety events (at 30 days or the end of index hospitalization):

In-hospital all-cause mortality.

Stroke.

VARC type 2-4 bleeding.

Major vascular, access-related, or cardiac structural complications.

Kidney injury stage 3 or 4.

Moderate or severe AR.

New permanent pacemaker due to procedure-related conduction abnormalities.

Surgery or intervention related to the device.

S5. Qualitative Image Quality Assessment

As a part of an additional study, within our cohort, a subset of 117 consecutive patients was used for quantitative analysis.

Measurements were performed on a dedicated workstation (Siemens Syngo.Via, Ve. B30, Erlangen, Germany) using TAVI planning software. The quantitative assessment included measurements of attenuation in Hounsfield Units (HUs) and the calculation of the signal-to-noise ratio (SNR). Additionally, the attenuation of adjacent muscle (pectoralis or rectus) was measured, and based on these measurements, the contrast-to-noise ratio (CNR) was calculated. Attenuation measurements in blood vessels were taken at the following heights: the aortic root, ascending and descending aorta (at the level of the main pulmonary artery), the intrathoracic aorta (at the level of the renal arteries), and the right common iliac and femoral arteries. Measurements were taken using the largest possible region of interest (ROI) for the measured vessel. CNR above three was considered diagnostic.

The prospective ECG-triggered scan group included 95 patients. The retrospective ECG-gated scan group comprised 22 patients. The average Body Mass Index (BMI) in the prospective ECG-triggered scan group was 27 (18-42±4.3), while in the retrospective ECG-gated scan group it was

31.3 (20.7-46.3±8.1). The difference in BMI was significant ($p<0.05$). The average weight in the prospective ECG-triggered scan group was 70 kg (SD ±13), and in the retrospective ECG-gated scan group, it was 80 kg (SD ±24), with no significant difference.

Attenuation values: Measurements of attenuation values in blood vessels in both groups, prospective ECG-triggered and retrospective ECG-gated, showed no significant differences. At all heights (aortic root, ascending and descending aorta, brachiocephalic, common iliac, and femoral arteries on the right side), the mean attenuation was above 300 HU (with an average range of 301-350 HU in the prospective ECG-triggered scan group and 345-449 HU in the retrospective ECG-gated scan group). In the prospective ECG-triggered scan group, only three measurements were below 100 HU: 81, 55, and 41 HU in three patients in the femoral arteries.

SNR values: SNR values in both groups at all measured heights showed no significant differences, ranging between average values of 19 to 28 in the prospective ECG-triggered scan group and 17 to 28 in the retrospective ECG-gated scan group.

CNR values: Average CNR values in the prospective ECG-triggered scan group for the aortic root and ascending aorta were 16±6.8 and 17±6.9, respectively, compared to 15.6±11 and 16.5±11.7 in the retrospective ECG-gated scan group. However, the CNR values were significantly lower in the prospective ECG-triggered scan group in the aortic root and ascending aorta ($p<0.005$). There was no significant difference between the two groups in CNR values at all other heights.
